# Ocular fundus pulsations within the posterior rat eye: Chorioscleral motion and response to elevated intraocular pressure

**DOI:** 10.1038/s41598-017-09310-1

**Published:** 2017-08-18

**Authors:** Marco Augustin, Stanislava Fialová, Corinna Fischak, Leopold Schmetterer, Christoph K. Hitzenberger, Bernhard Baumann

**Affiliations:** 1 Medical University of Vienna, Center for Medical Physics and Biomedical Engineering, 1090 Vienna, Austria; 2 General Hospital and Medical University of Vienna, Department of Clinical Pharmacology, 1090 Vienna, Austria; 3 Singapore Eye Research Institute, The Academia, 169856 Singapore, Republic of Singapore; 4 Lee Kong Chian School of Medicine, Nanyang Technological University, 308232 Singapore, Republic of Singapore

## Abstract

A multi-functional optical coherence tomography (OCT) approach is presented to determine ocular fundus pulsations as an axial displacement between the retina and the chorioscleral complex in the albino rat eye. By combining optical coherence elastography and OCT angiography (OCTA), we measure subtle deformations in the nanometer range within the eye and simultaneously map retinal and choroidal perfusion. The conventional OCT reflectivity contrast serves as a backbone to segment the retina and to define several slabs which are subsequently used for quantitative ocular pulsation measurements as well as for a qualitative exploration of the multi-functional OCT image data. The proposed concept is applied in healthy albino rats as well as in rats under acute elevation of the intraocular pressure (IOP). The evaluation of this experiment revealed an increased pulsatility and deformation between the retinal and chorioscleral complex while increasing the IOP level from 15 mmHg to 65 mmHg. At IOP levels exceeding 65 mmHg, the pulsatility decreased significantly and retinal as well as choroidal perfusion vanished in OCTA. Furthermore, the evaluation of the multi-parametric experiment revealed a spatial correlation between fundus pulsatility and choroidal blood flow. This indicates that the assessed pulsatility may be a valuable parameter describing the choroidal perfusion.

## Introduction

Optical coherence tomography (OCT) is a real-time, non-invasive optical imaging method for measuring translucent or semi-translucent samples with micrometer resolution. Since its introduction 25 years ago^1^, advances in imaging speed, resolution and functionality made OCT a standard diagnostic tool for clinical ophthalmologists^2^. Apart from its application in ophthalmology, OCT has been applied, among other disciplines, in dermatology^3^, cardiology^4^ as well as in neurology and cancer research in general^5^.

In conventional OCT the contrast is based on the intensity of the light backscattered and reflected by the sample at various depths along the probing beam. Thus, OCT provides detailed morphological information by scanning the beam over the sample and reconstructing its three-dimensional (3D) structure. Functional extensions of OCT were developed by exploiting the polarization state of the backscattered light - in polarization-sensitive (PS-OCT)^6–8^ - or by modifying scanning patterns to enable the application of motion contrast algorithms - in OCT angiography (OCTA)^9–11^. Furthermore, the phase information incorporated in the OCT signal can be used to detect subtle relative motions between successive scans which is for example used in Doppler (D)OCT^12^ to measure blood flow. Phase-differences between consecutive depth profiles (A-scans) or tomograms (B-scans) can also be used to determine relative displacement and sample deformation in optical coherence elastography (OCE)^13–15^. Traditionally, in OCE a load is used to cause a deformation of the imaged sample and the measured pulse propagation is correlated to the impulse function to derive biomechanical properties, such as stiffness or elastic modulus^15^. In ophthalmology, OCE was applied in the past to investigate various ocular structures *ex-vivo* ^16–18^ as well as *in-vivo* ^19–24^.

The ocular in- and outflow of blood induces volumetric changes to the eye ball and thus movement of ocular structures and a variation of the intraocular pressure (IOP) synchronous with the cardiac cycle^25^. First methods to detect these pulsatile changes in IOP and displacement were demonstrated using pneumotonometry^25^ and laser interferometry^26, 27^, respectively. Recently, Fourier-domain (FD) OCT was used for measuring pulse-induced deformations between the cornea and the retina^28^, within the anterior chamber^20, 23^, as well as within the posterior eye^19, 21^. In the past, ocular fundus pulsations were associated with the elasticity and thus the stiffness and rigidity of ocular tissues^29^. Elevated IOP is the most important risk factor for glaucoma and assumed to play a major role in the pathogenesis of the disease. Hence, IOP is a key parameter in the diagnosis and treatment of patients suffering from glaucoma. IOP changes are associated with alterations of the biomechanical properties of the eye and various clinical and preclinical studies were performed to study this relation^30–33^. While clinical studies are limited to the actual physiological and pathological states of volunteers and patients, preclinical studies allow to investigate an extended range of scenarios, e.g. artificial elevation of IOP to different levels.

A popular approach to such investigations in rodents is to artificially increase the IOP by cannulation of the eye in preclinical settings^34–37^. OCT, adapted for small animal imaging, and its functional extensions can hereby be used to assess morphological and physiological parameters of the rat eye during an acute increase of IOP *in vivo* ^38, 39^. Hence, conventional OCT was used to study reflectivity and thickness changes in the retina^40, 41^ and OCTA as well as DOCT were used to study the perfusion of the posterior eye as a response to an elevated IOP^35, 42^. Recently, our group adapted PS-OCT for small animal imaging and demonstrated its benefits in healthy mice and rats^43–45^. Consequently, we recently used a PS-OCT system to study the optical and structural properties of the posterior rat eye as a response to an acute elevation of the IOP^34^.

In this work, we extend the analysis of our recent acute IOP study and propose (i) a method to map and measure the ocular fundus pulsations in the posterior rat eye by high-speed spectral domain (SD) OCT and (ii) combine it with OCTA to simultaneously assess perfusion parameters. The proposed multi-functional OCT imaging approach is consequently used (iii) to study the relation between elevated IOP, fundus pulsations and retinal perfusion in the posterior rat eye.

## Methods

### Multi-functional OCT image acquisition

An OCT system tailored for small animal retinal imaging was utilized in this work. The system was previously described in detail by Fialová *et al*.^45^. In brief, a PS-OCT imaging system featuring a superluminescent diode centered at ${\lambda }_{c}=\mathrm{840\ }nm$ (${\rm{\Delta }}\lambda =\mathrm{100\ }nm$) along with custom-built spectrometers was assembled. The system was operating at an A-scan rate of 83 kHz, provided an axial resolution of approximately $\mathrm{3.8\ }\mu m$ and the sample arm was designed such that a field of view of 30° × 30°, corresponding to approximately $\mathrm{1.5\ }mm\times \mathrm{1.5\ }mm$ on the rat retina, could be scanned. An overview of the multi-functional post-processing pipeline which enables motion contrast based OCTA, PS contrast along with reflectivity contrast and a graph-based retinal layer segmentation was described previously^46^. Volumetric data was acquired comprising 512 A-scans per B-scan with five B-scan repetitions (MB) at 400 distinct positions (NB). The acquisition time was approximately 15 s with an inter B-scan time ${\tau }_{B}\,\mathrm{=7.65\ }ms$ (130 Hz frame rate).

### Determination of fundus pulsations

**Figure 1:**
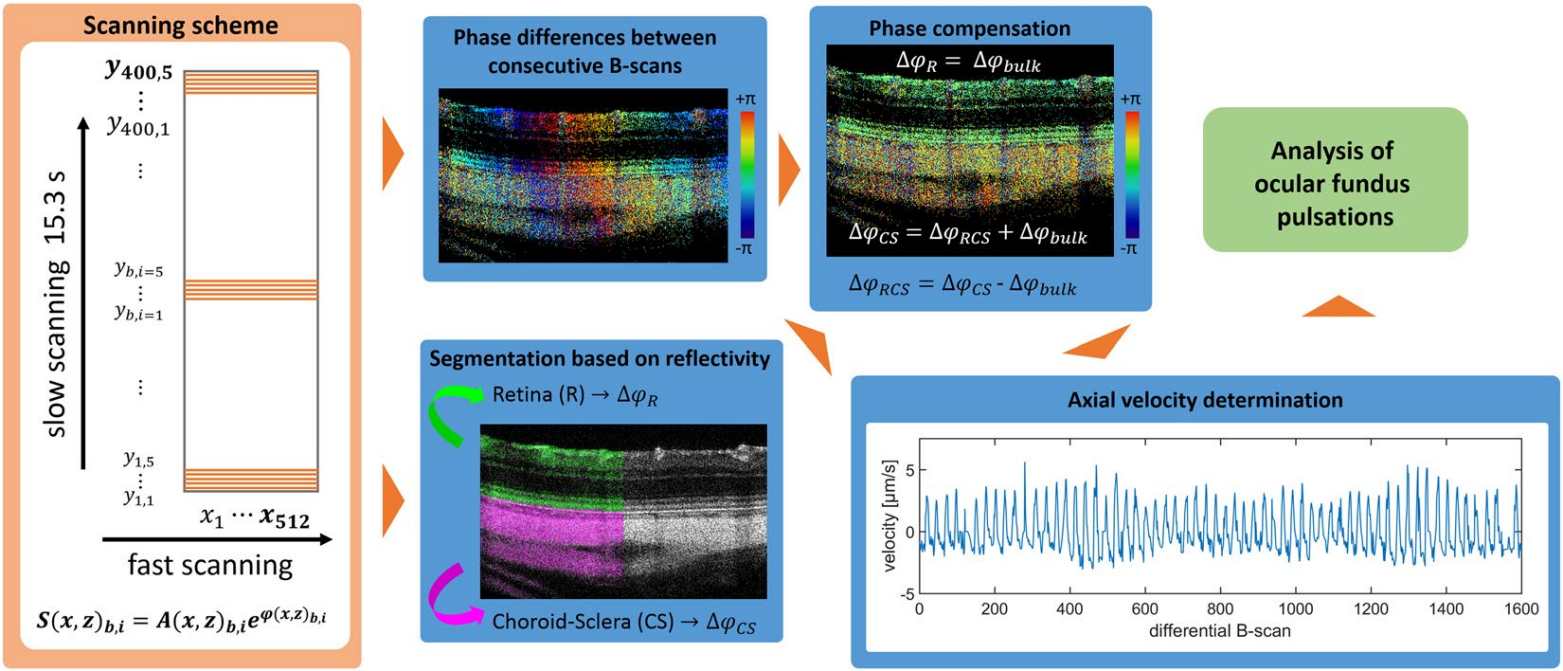
Overview of the ocular fundus pulsation measurement. A repeated raster scanning pattern was used to perform phase-differencing between consecutive B-scans. After segmenting the retinal and chorioscleral region, bulk phase shifts (estimated in the retina) were subtracted from each B-scan and the remaining average phase differences between the retina and chorioscleral complex were calculated. The axial velocities of these deformations were subsequently used for a quantitative spectral analysis.

The ocular fundus pulsations were assessed by exploiting the time lapse between two consecutive frames. The differential phase images were hereby divided into a retinal (R) and chorioscleral (CS) region based on the retinal pigment epithelium (RPE) segmentation of the structural OCT information. The deformation between these two regions was determined as the average axial phase shift between R and CS within one A-scan. An overview of the proposed fundus pulsation determination method is depicted in Fig. 1 and the proposed algorithm is described in the following. Each pixel in a B-scan is represented by a complex-valued signal S comprising amplitude A and phase *φ* at the position x (fast scanning direction) and z (depth).

1 $${S}_{b,i}(x,z)={A}_{b,i}(x,z)\cdot {e}^{j\cdot {\phi }_{b,i}(x,z)}\mathrm{,\ }b=1\cdots NB\mathrm{,\ }i=1\cdots MB$$

Phase differences ${\rm{\Delta }}{\phi }_{b,i}$ are calculated between consecutive B-scans in each set of MB repetitions as

2 $${\rm{\Delta }}{S}_{b,i}(x,z)={S}_{b,i}(x,z)\cdot {S}_{b,i+1}{(x,z)}^{\ast }\mathrm{,\ }b=1\cdots NB\mathrm{,\ }i=1\cdots MB-1$$

3 $${\rm{\Delta }}{\phi }_{b,i}(x,z)=arg({\rm{\Delta }}{S}_{b,i}(x,z))\mathrm{,\ }b=1\cdots NB\mathrm{,\ }i=1\cdots MB-1$$

where * denotes the conjugate complex value and *arg* the argument. To compensate for phase drifts during a differential B-scan due to bulk motion, a phase difference term $\Delta {\phi }_{bulk}(x)$ was estimated as the argument of the complex vector sum along the z-direction for each A-scan position x in the retinal (R) part of the posterior rat eye, where ${z}_{R}$ and ${z}_{I}$ were the coordinates of the RPE and the inner limiting membrane (ILM), respectively:

4 $${\rm{\Delta }}{\phi }_{bulk}(x)=arg(\sum _{z={z}_{I}}^{{z}_{R}}{\rm{\Delta }}{S}_{b,i}(x)).$$

Here by, phase information was only used for pixels exceeding a threshold value in the corresponding reflectivity images^46^. With the retina serving as a reference, the retinal and chorioscleral relative phase differences ${\rm{\Delta }}{\phi }_{R}(x)$ and ${\rm{\Delta }}{\phi }_{CS}(x)$ can be expressed as

5 $${\rm{\Delta }}{\phi }_{R}(x)={\rm{\Delta }}{\phi }_{bulk}(x)$$

6 $${\rm{\Delta }}{\phi }_{CS}(x)={\rm{\Delta }}{\phi }_{RCS}(x)+{\rm{\Delta }}{\phi }_{bulk}(x)$$

where the phase difference ${\rm{\Delta }}{\phi }_{RCS}(x)$, encountered between the retina and the chorioscleral region, i.e. the phase shift induced by chorioscleral pulsatility relative to the retina, was calculated:

7 $${\rm{\Delta }}{\phi }_{RCS}(x)={\rm{\Delta }}{\phi }_{CS}(x)-{\rm{\Delta }}{\phi }_{bulk}(x).$$

The axial velocity $v$ of the fundus pulsation was then determined as

8 $$v(x)=\frac{{\rm{\Delta }}{\phi }_{RCS}(x)\cdot {\lambda }_{c}}{4\cdot \pi \cdot n\cdot {\tau }_{B}}$$

and was limited by the central wavelength of the system ${\lambda }_{c}$ and the inter B-frame time ${\tau }_{B}$. The refractive index n was assumed to equal 1.35. With the settings used in this work, an axial velocity range of $[-20.33\,\mu m/s,+20.33\,\mu m/s]$ was determined. Equation 8 provides the axial velocity component, i.e. fundus motion parallel to the OCT beam. To correct for the shape of the retina, and thus to reconstruct pulsatile motion perpendicular to the fundus, a correction value was determined and used as a weight for the phase differences ${\rm{\Delta }}{\phi }_{RCS}$. For this purpose, the retinal shape was approximated along each A-scan as previously described for motion compensation and flattening^46^. Consequently, the retinal surface was smoothed by a polynomial fit and its surface normals $g=[{g}_{x},{g}_{y},{g}_{z}]$ were determined. The angle *α* between *g* and the beam direction $[\mathrm{0,0,1}]$ was calculated and the velocity was corrected to read

9 $${v}_{RCS}=\frac{v}{\cos (\alpha )}$$

for every pixel. The developed method compensates solely for axial bulk motion and hence no compensation for transversal or rotational eye movements was implemented. Nevertheless, in case of severe axial or transversal motion causing uncorrelated phase differences, the respective velocity values were excluded and substituted by linear interpolation.

### Quantitative analysis of ocular fundus pulsations

**Figure 2:**
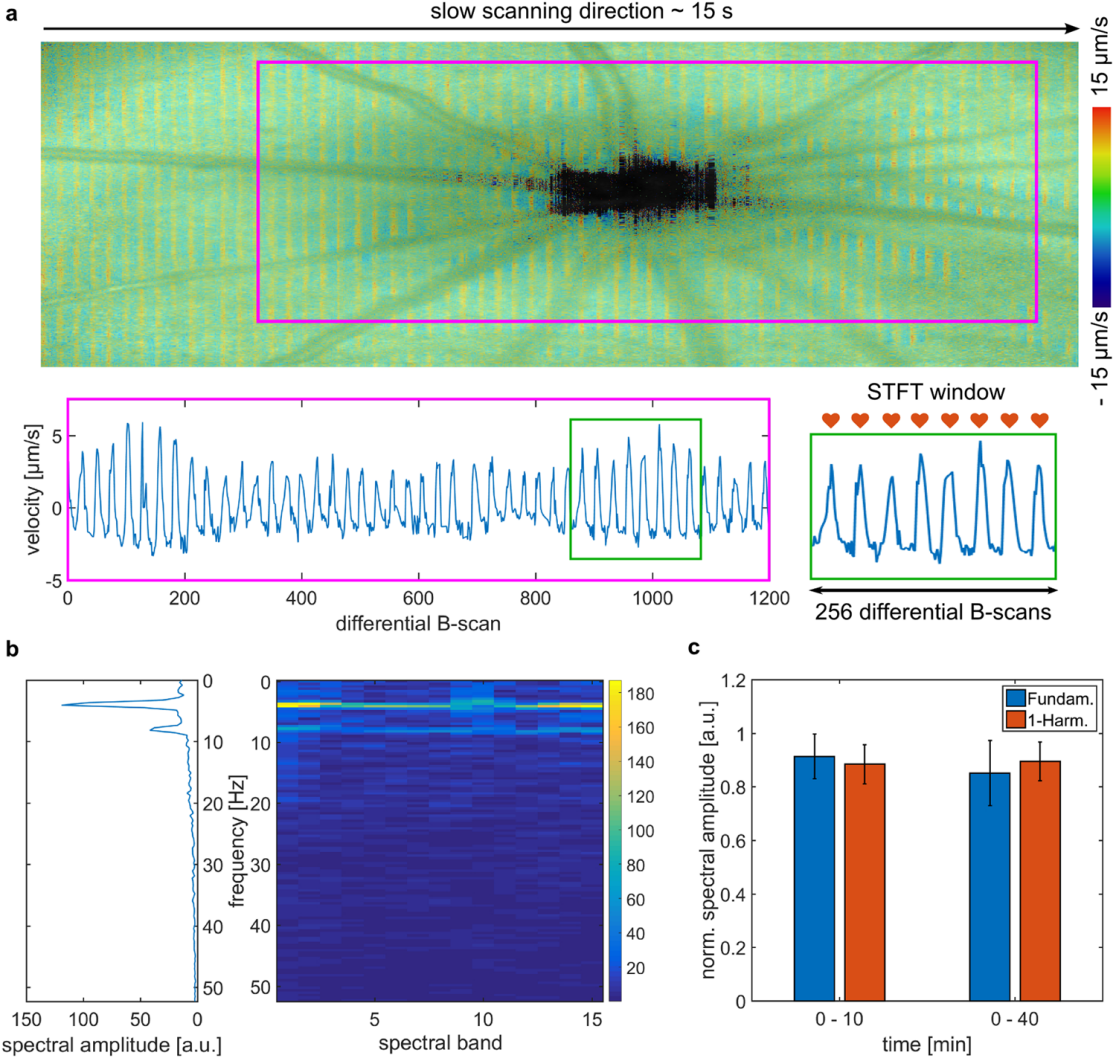
Fundus pulsation analysis and repeatability measurements. (**a**) An exemplary fundus pulsation map overlaid on the corresponding reflectivity en-face projection at physiological IOP. Fundus pulsations were determined as velocity between retinal and chorioscleral complex. The velocities were averaged for each B-scan and subsequently analyzed by a STFT within a sliding window depicted in green. (**b**) The spectrogram showing the 15 spectral bands determined by the STFT and average spectral amplitude for the fundus pulsations shown in (**a**). The average spectral amplitudes were calculated for the fundamental oscillation as well as for the first harmonic. (**c**) The repeatability was investigated in a short time range of approximately 10 minutes (3 measurements each) as well as on a longer time span of approximately 40 minutes (total of 5 measurements each) for three rats. The normalized mean spectral amplitudes of the fundamental oscillation as well as of the first harmonic are shown, where error bars indicate the standard deviation.

One approach to analyze pulse waveforms is based on Fourier analysis, where the repeating pulse function is decomposed into its sinusoidal waveform components^28, 31, 47^. The frequency component of the highest spectral power (fundamental oscillation) in the Fourier analysis is representing the arterial pressure pulse while higher order oscillations at integer multiples of the fundamental oscillation (so-called harmonics) are related to other parameters in cardiovascular research such as the vessel’s vascular impedance^48^. To quantify the ocular fundus pulsations as a response to increased IOP a spectral analysis of the pulsatile motion was performed. For this purpose, the average velocity $\overline{{v}_{RCS}}$ within each B-scan was determined and analyzed using a short-time Fourier transform (STFT). The window of the STFT was set to 256 B-scans and thus including approximately 10 heart cycles (at a heart rate of 350 bpm) with an empirically chosen overlap of 75% (as indicated in Fig. 2(b)). The resulting 15 spectra per volume were averaged and the amplitude of the fundamental oscillation as well as its first harmonic was calculated. To overcome small variations in the imaging position during the experiment, the center of the optic nerve head (ONH) was specified manually and a region of approximately 1.15 mm × 1.15 mm, comprising 400 A-scans (x) and 1200 differential B-scans (y), was used for evaluation.

### OCTA analysis

OCTA was used to assess retinal and choroidal blood flow during the experiment. Based on the multi-functional image processing pipeline proposed in our previous work^46^, the ocular vascular plexuses^49, 50^ were differentiated by the retinal layer segmentation. In this work, three plexuses were defined in the posterior rat eye:

- Plexus 1- Superficial retinal capillary plexus (SCP): Larger vessels in the retinal nerve fiber layer (RNFL), the ganglion cell layer (GCL) and connective vessels in the inner plexiform layer (IPL).
- Plexus 2- Deeper retinal capillary plexus (DCP): Capillary blood vessel network in the outer plexiform layer (OPL).
- Plexus 3- Chorioscleral (CS) plexus: Vessels in the choroid and sclera.

En-face projections of the plexuses were analyzed by calculating the flux, which was defined as the mean flow signal intensity^51^ in the respective blood flow regions. The Frangi vesselness filter^52^ was used to enhance vascular structures in the SCP and DCP angiograms prior to calculating the flux^51^. Larger vessels in the SCP were segmented and respective areas were excluded before determining the flux in plexus 2 and 3 to avoid the influence of vessel shadowing artefacts. The flux was determined in the evaluation region of 400 A-scans and 1200 B-scans mentioned before, thereby avoiding a circular area centered at the ONH with a diameter of 150 pixels (440 *μm*).

### Animals and IOP experiment

Eight adult, male Sprague Dawley rats (427 ±68 g) were purchased from the Medical University of Vienna breeding facility, Himberg, Austria. The animals were kept under controlled lighting conditions (12 hours dark, 12 hours light) with food and water ad libitum. The preparation of the animals and the execution of the experiment were described in detail by Fialová *et al*.^34^. In short, the animals were anesthetized using midazolam (Dormicum, Roche, Austria GmbH, Vienna, Austria, 1.0 ml/kg body weight), medetomidine (Domitor, Bayer Austria GmbH, Vienna, Austria, 0.2 ml/kg body weight) and fentanyl (Fentanyl, Hameln Pharma Plus GmbH, Hameln, Germany, 0.3 ml/kg body weight) by intra-peritoneal injection. The rats were placed in a custom-made animal stage and the head was fixated with cushioning material and medical tape. The anterior chamber of the animal’s right eye was cannulated with a needle connected to a reservoir containing a 0.9% NaCl solution via a pressure transducer. The transducer was positioned at the same height as the eye and used to determine the pressure level. Stepwise increase of the IOP level from 15 mmHg to 105 mmHg was accomplished by lifting the reservoir. OCT measurements were performed approximately every 5 minutes with a total duration of one hour per animal (excluding preperation). At the endpoint, the rats were sacrificed by an overdose of sodium pentobarbital (Release, Richter Pharma AG, Wels, Austria). All experiments were performed in accordance with the Association for Research in Vision and Ophthalmology Statement for the Use of Animals in Ophthalmic and Vision Research and under a protocol approved by the institutional ethics committee and the Austrian Federal Ministry of Science, Research and Economy (protocol number GZ 66.009/0005-WF/V/3b/2016).

### Statistical analysis

A statistical analysis was performed for all quantitative parameters determined during the experiment. A non-parametric Friedman test with post-hoc Dunn-Bonferroni-tests for pairwise comparisons between different IOP levels was computed. All p-values were Bonferroni corrected and a p-value < 0.05 was defined as statistically significant. Results in the manuscript are given as mean ± standard deviation unless stated otherwise.

### Data Availability

The datasets generated during and analysed during the current study are available from the corresponding author on reasonable request.

## Results

### Validation of proposed fundus pulsations method

The repeatability of fundus pulsations measurements was investigated in three healthy Sprague-Dawley rats at their physiological IOP level, i.e. without cannulation of the eye. For this purpose, three repeated measurements were taken in a short time window of t = [0, 6, 11] minutes and two additional measurements at t = [26, 38] minutes for each rat. The heart rate of the rat was measured with a pulse oximeter (STARR Life Sciences MouseOx Plus) attached to one of the hind limbs. In Fig. 2(a) a fundus pulsation map with averaged axial velocities overlaid on the en-face reflectivity image is shown. A striped pattern along the slow scanning direction can be observed as a result of the ocular fundus pulsations. The average heart rate determined by the OCT fundus pulsation method was 228 (±12) bpm which is in good agreement with the measurements during the OCT experiment using the pulse oximeter, 239 (±4) bpm. The average velocity for each B-scan was determined in the rectangular evaluation window centered at the ONH (pink rectangle) and is depicted in the plot below. The fundus pulsations corresponding to the axial velocities were analyzed using a STFT with a sliding window of the size depicted by a green rectangle in the plot, resulting in a total of 15 spectral bands. The spectrogram from the STFT is depicted in Fig. 2(b) together with the averaged spectral response for the 15 bands. The spectral analysis was performed for the three rats at the five points and the spectral responses were normalized to the maximum response for each rat. A bar plot showing the mean and standard deviation for the fundamental as well as for the first harmonic for the two time intervals is shown in Fig. 2(c), for all rats. The coefficient of variation for the fundamental amplitude was determined with 9.2% and 14.2% for the short and the total time of the experiment, respectively. For the first harmonic the coefficient of variations was determined with 8.2% and 8.0% for the short and the total time of the experiment, respectively.

### Spectral analysis of fundus pulsations in response to elevated IOP

**Figure 3:**
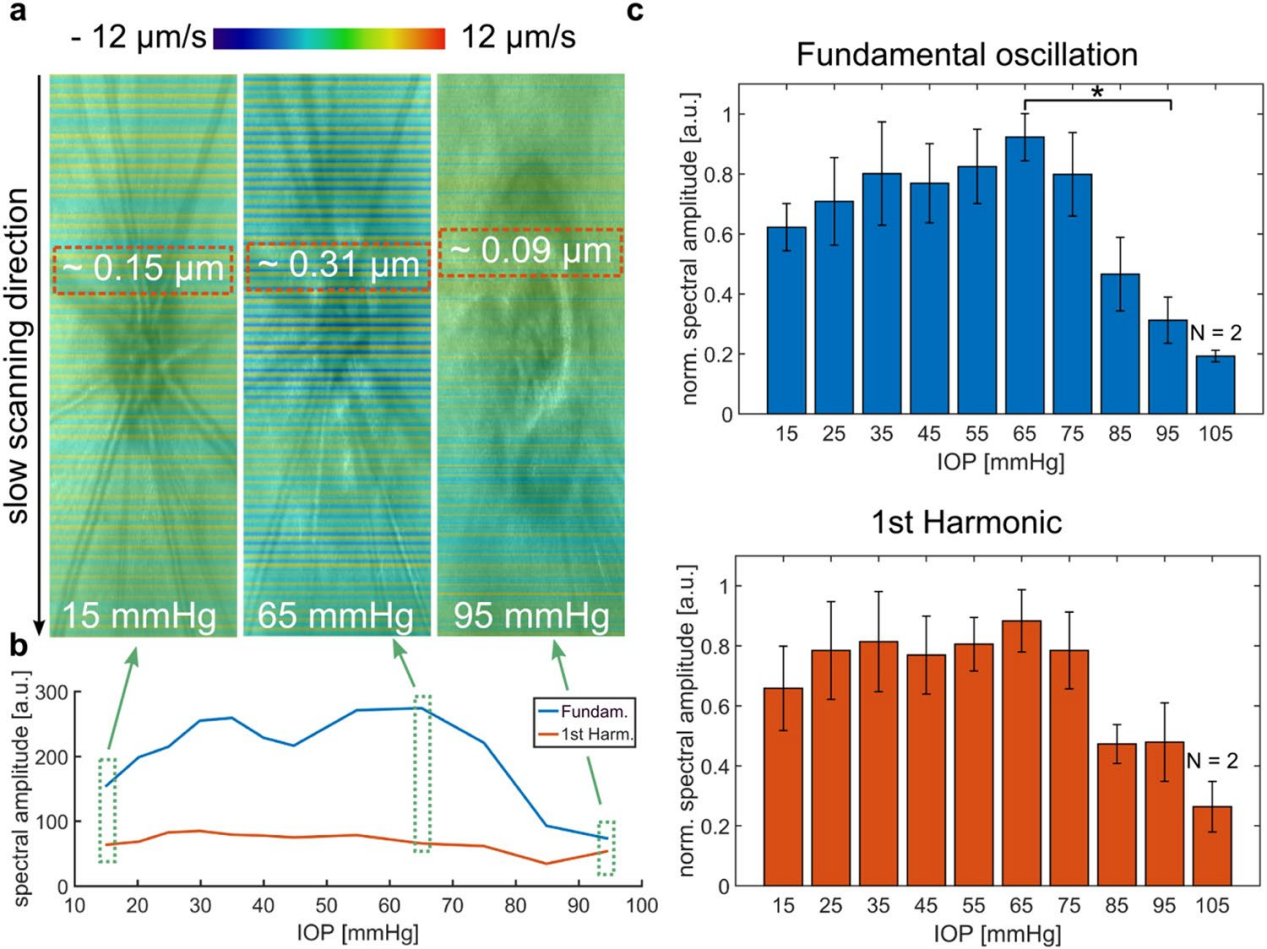
Spectral analysis of ocular fundus pulsations as a response to an elevated IOP. (**a**) Pulsation changes illustrated as a color-coded overlay on the en-face reflectivity projections at three different IOP levels (15, 65, and 95 mmHg). A stronger stripy pattern, in comparison to the initial IOP level, was noticed at IOP of 65 mmHg while the pulsations almost vanished at 95 mmHg. (**b**) Pulsatile response to an increased IOP shown for one rat exemplary. (**c**) Pulsatile response to an elevated IOP for four rats (N = 4) in a range of 15 to 105 mmHg. Error bars indicate the standard deviation. The spectral amplitude was normalized for each rat to the maximum spectral response. A significant drop was noticed between 65 mmHg and 95 mmHg in the fundamental osciallation. The analysis of the first harmonic showed a similar behavior, but no statistically significant trend was observed. (*significant difference: *p* < 0.05).

Fundus pulsation analysis was performed for five animals at IOP levels ranging from 15 to 95 or 105 mmHg, where retinal perfusion stopped. One animal did not show a substantial reduction in retinal perfusion at high IOP, possible due to a pressure drop between the transducer and the eye (e.g. bad cannulation). Hence, the measurement for this animal was excluded from all of the subsequent quantitative analysis. Figure 3(a,b) shows an exemplary quantitative evaluation of the fundus pulsations as a response to increased IOP. The qualitative representation of the fundus pulsation overlaid on the corresponding reflectivity en-face image in Fig. 3(a) reveals a more prominent stripy pattern at IOP increased to 65 mmHg before pulsations vanished at 95 mmHg. In Fig. 3(b) the spectral amplitudes of the fundamental oscillation and the first two harmonics are shown for different IOP levels. For the rat shown in Fig. 3(a), the absolute displacement averaged over five cardiac cycles in the proximity of the ONH (position is indicated by red dotted rectangle) was 150 nm, 300 nm and 90 nm for the IOP levels of 15 mmHg, 65 mmHg and 95 mmHg, respectively. The trend of an increase in the power of the pulsations before dropping at 65 mmHg was observed in all animals. Figure 3(c) shows the average normalized spectral amplitude and its standard deviation for the fundamental oscillation as well as the first harmonic. The spectral fundus pulsation response peaked at 65 mmHg before it substantially dropped until 95 mmHg. A statistically significant drop was only noticed between IOP levels of 65 mmHg and 95 mmHg, respectively (p = 0.018). A similar trend was noticed for the analysis of the first harmonic, however no statistical significance was observed.

### IOP dependent perfusion changes

**Figure 4:**
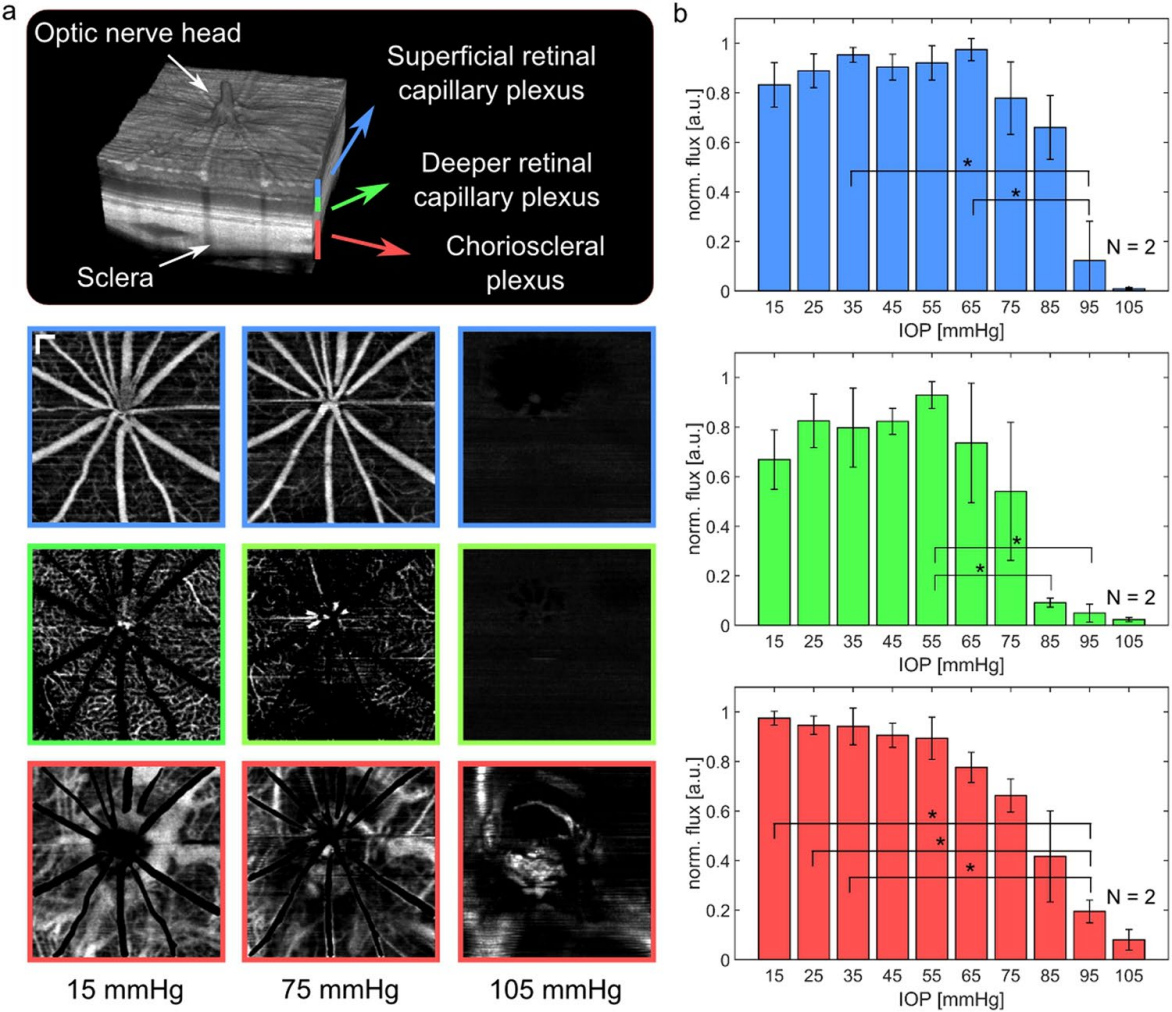
Changes of the retinal perfusion in response to increased IOP. (**a**) En-face OCTA maximum-intensity-projections of three plexuses at three different IOP levels. Non-perfused patches in the deeper retinal capillary plexus were observed at increased IOP levels of 75 mmHg. While the retinal perfusion stopped at an IOP level of 105 mmHg, some major choroidal vessels were still present in the OCTA images. (scale bar = 150 *μm*) (**b**) Quantitative analysis of the flux in all three plexuses in four animals. The flux in the superficial retinal plexus dropped significantly in the range of 65 to 95 mmHg while the flux in the deeper retinal capillary plexus earlier decreased significantly between 55 mmHg and 85 mmHg. (*significant difference: *p* < 0.05).

To investigate the correlation between the fundus pulsations changes and perfusion, OCTA was performed. Figure 4(a) shows the response of the different plexuses to elevated IOP. While the superficial plexus did not appear substantially different at 65 mmHg, a more severe change in the deeper retinal capillary plexus can be noticed. No retinal perfusion (plexus 1 and 2) was observed at an IOP of 105 mmHg, however, some large choroidal vessels were still present in the OCTA images. The evaluation of the flux is shown in Fig. 4(b) for all animals, where the flux measurements were normalized by the maximum detected flux for each animal individually. Blood flux in the superficial plexus exhibited no substantial change until 65 mmHg. Beyond 75 mmHg smaller connective vessels vanished before a significant reduction of retinal perfusion was encountered at 95 mmHg, see Fig. 4(b). In contrast, the flux in the deeper retinal plexus showed a decreasing trend starting with 55 mmHg and was significantly lower at 85 mmHg. The calculated flux in the choroidal plexus showed a more steady decrease when compared to the retinal plexuses. Significant changes were determined between the IOP of 95 mmHg and 15 mmHg, 25 mmHg and 35 mmHg, respectively.

### Locations of increased pulsatile motion at elevated IOP levels

**Figure 5:**
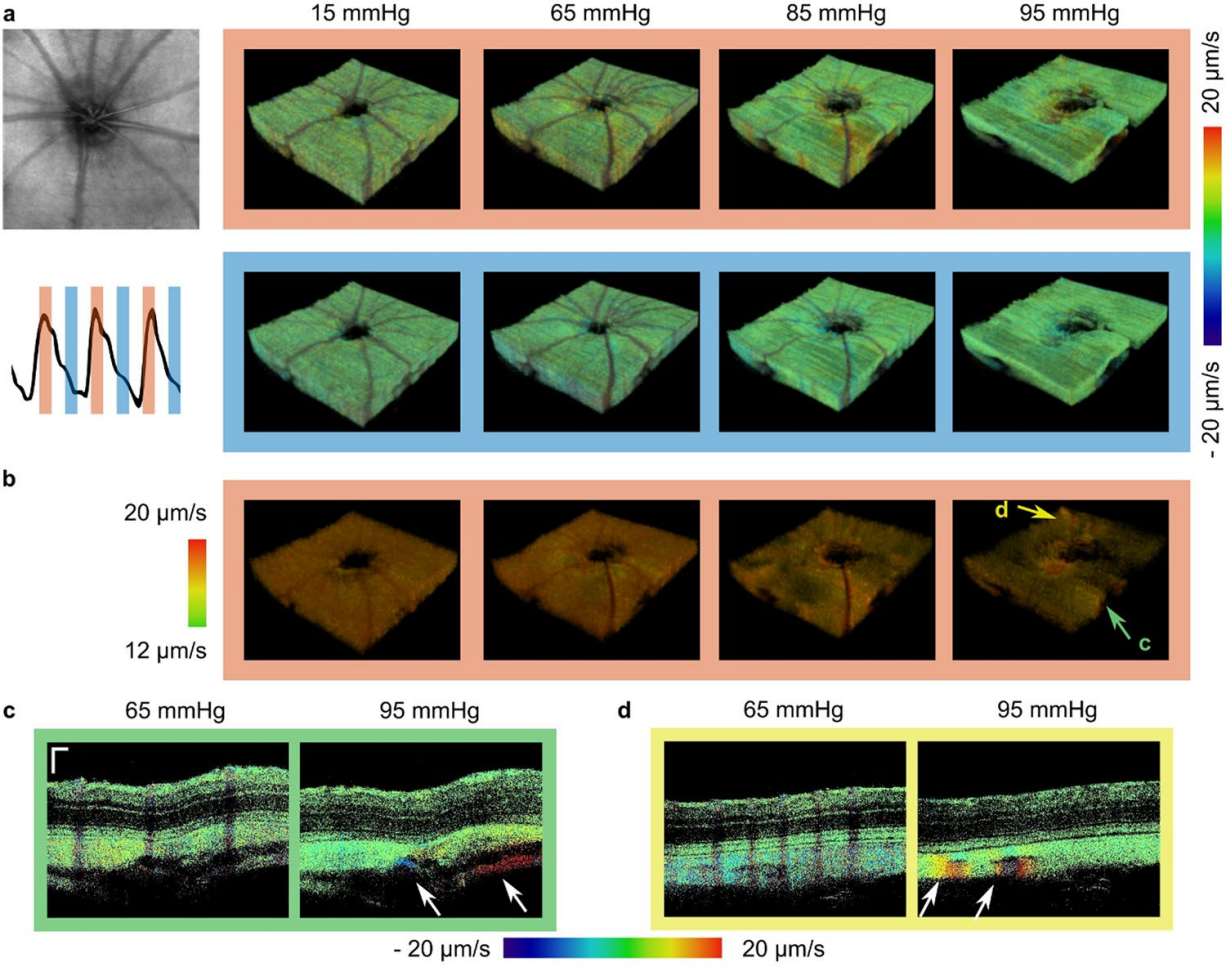
Time gated visualization of ocular fundus pulsations. (**a**) The recorded volumes were time gated according to the heart cycle for visualization. The top row shows 3D renderings of the CS complex at four different IOP levels ranging from 15 mmHg to 95 mmHg at the instance of maximum deformation. The bottom row shows the corresponding volumes at half a heart cycle later. (**b**) Volumes are shown for motions exceeding 12 *μm*/*s* for the same datasets as shown in (**a**). Locally increased deformations were identified at higher IOP levels while the overall pulsatility decreased with increased IOP above 65 mmHg. (**c**,**d**) B-scans at the locations indicated by arrows in (**b**) are shown at IOPs of 65 mmHg and 95 mmHg, respectively. Regions undergoing increased deformations were identified around the ONH and larger choroidal vessels. (scale bar = 100 *μm*).

To identify regions undergoing similar deformations, the recorded images were time gated according to the heart cycle as depicted in Fig. 5(a). The heart cycle was therefore divided into 20 sections of equal duration from which three adjacent sections were used for averaging and subsequent visualization. Three-dimensional renderings of the CS complex are shown in Fig. 5(a) for two of these sections at four IOP levels ranging from 15 mmHg to 105 mmHg. Increased local deformations can be observed at higher IOP levels of 85 mmHg and 95 mmHg and are even better visible in the thresholded volumes where only regions exceeding an axial velocity of 12 *μm*/*s* are shown, see Fig. 5(b). While only a moderate, uniform pulsation in the chorioscleral complex was observed at 15 mmHg, the increase in pulsation power becomes more pronounced at 65 mmHg (see Supplementary Video 1). At higher IOP levels, the pulsation vanished and deformations were identified only in distinct regions. Two of these regions are highlighted in Fig. 5(b), B-scans at two different IOP levels are shown in Fig. 5(c,d), respectively. These regions are associated with larger choroidal blood vessels close to the ONH (Fig. 5(c)) but also superior to the ONH (Fig. 5(d)). The B-scans taken at the same phase of the cardiac cycle reveal a local increase of deformation when increasing the IOP level from 65 mmHg to 95 mmHg.

### Extended field-of-view fundus pulsation map at physiological IOP

**Figure 6:**
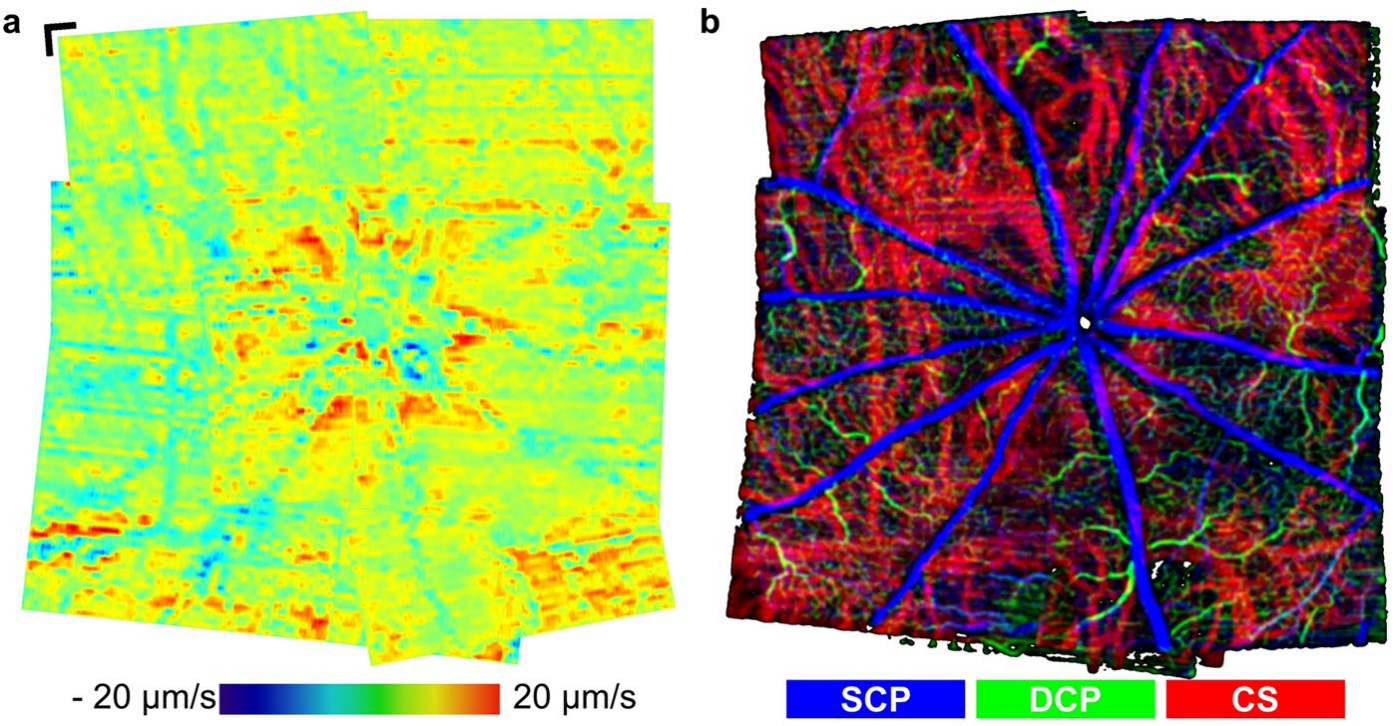
Extended field-of-view fundus pulsation maps and OCTA at physiological IOP. (**a**) Pronounced deformations were encountered around the ONH and close to major choroidal blood vessels in some peripheral regions. A time lapse of time gated fundus pulsations along one heart cycle is shown in Supplementary Video 2. (**b**) Extended field-of-view OCTA image. The depth positions of the three plexuses are color coded in this OCTA image. The superficial capillary plexus (SCP) containing the retinal nerve fiber layer, ganglion cell layer and the inner plexiform layer, the deeper retinal capillary plexus (DCP) in the outer plexiform layer as well as the chorioscleral (CS) plexus are shown in blue, green and red, respectively. Regions of increased pulsatility were identified as regions containing major blood vessels in the OCTA images of the choroid. (scale bar = 150 *μm*).

In order to further investigate the relation between fundus pulsations and choroidal blood perfusion in a larger extent of the posterior pole, nine datasets were acquired around the ONH of a control rat at physiological IOP and subsequently stitched to increase the field-of-view (FoV). Time gated en-face pulsation maps as well as OCTA maps were generated as shown in Fig. 6. A video of the time gated extended FoV pulsation map is provided in Supplementary Video 2. The peripapillary region exhibited increased fundus pulsations at the physiological IOP level. Furthermore, increased pulsatility was also observed in some peripheral regions close to major choroidal vessels.

## Discussion

Quantitative assessment of fundus pulsations enables the study of biomechanical properties of the eye. Biomechanical abnormalities of ocular tissue may trigger the long term development of ocular diseases such as glaucoma or myopia and hence are key in the understanding of the pathophysiology of these diseases. This work demonstrated the quantitative assessment of ocular fundus pulsations along with simultaneous OCTA in healthy rats at physiological IOP as well as in an experimental setup of acute IOP elevation in the posterior rat eye.

**Figure 7:**
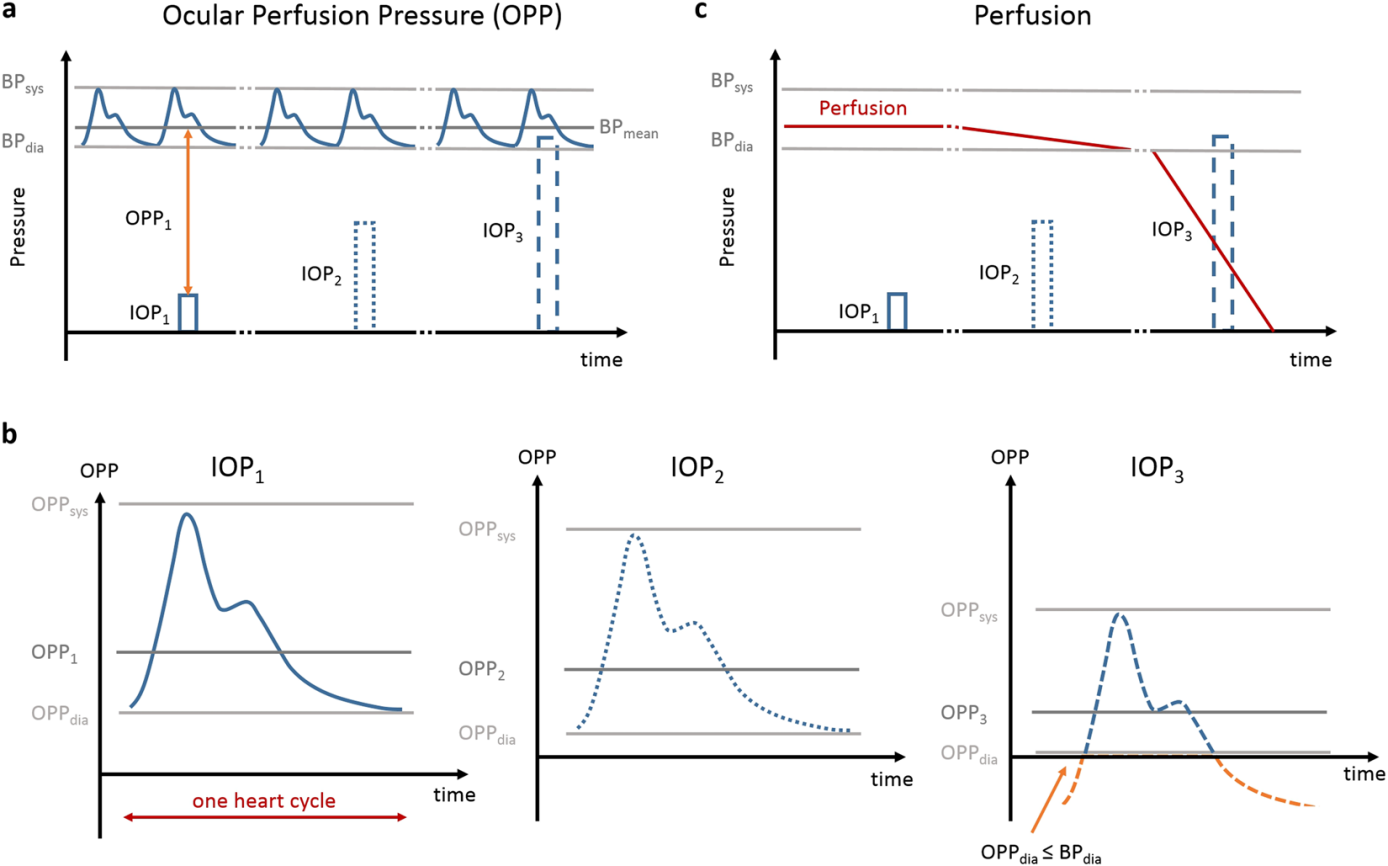
Model of the ocular perfusion pressure (OPP) and perfusion changes during IOP elevation. (**a**) An elevation of the IOP will cause a decrease in the mean OPP. (**b**) The pressure amplitude defined as the difference between the systolic and diastolic pressure level remains unchanged until the diastolic OPP undercuts the diastolic blood pressure. (**c**) The ocular perfusion will stop at this threshold and hence the fundus pulsations will vanish as well.

The latter experiment revealed a substantial increase of the fundus pulsations when IOP was increased until 65 mmHg. The ocular perfusion pressure (OPP) is defined as the difference between the arterial and venous blood pressure, where the venous blood pressure can be approximated by the IOP. Thus, an increase in IOP will lead to an decrease of the OPP^53^. At lower IOPs it is only the non-pulsatile component of the ocular perfusion pressure that is reduced. As such one would assume that the pulsatile component of ocular perfusion pressure stays constant until the diastolic pressure is reached. A significant drop in fundus pulsations was encountered between 65 mmHg and 95 mmHg. In this IOP range the diastolic blood pressure reaches the IOP level. Hence, the blood flow and the rhythmic filling of the eye ball will stop and result in a decrease of fundus pulsations. An explanation for the initial increase in pulsation power could be an increased stretch and accordingly an increased rigidity of the eye ball^54–56^. Thus, the volumetric change that occurs during the cardiac cycle and the rhythmic filling of vessels also causes stronger fundus pulsations. A model of the relation between the OPP amplitude and IOP changes is illustrated in Fig. 7(a,b). The IOP range of 65 mmHg in which the drop in fundus pulsatility was encountered is in good agreement with the diastolic blood pressure level of anaesthetized rats with a mean blood pressure level of around 90 mmHg^57^. When the IOP level was raised above the diastolic blood pressure, some remaining pulsations were observed, most likely due to residual movement of blood caused by heart beat activity. Another phenomenon that needs to be considered is potential choroidal autoregulation^58, 59^, which may lead to changes in vascular resistance during the IOP increase. The evaluation of the acute IOP elevation experiment proved the ability of the proposed method to assess physiological phenomena related to the dynamics of blood flow as well as elastic characteristics.

The quantitative analysis of the OCTA data showed a trend of increasing flux, interpreted by the decorrelation amplitude, until 65 mmHg and 55 mmHg in the superficial and deeper retinal capillary plexus, respectively. The interpretation of the flux values has to be made with caution as the dynamic range of the OCTA decorrelation signals is rather low due to the relatively long B-scan interval of 7.65 ms. On one hand, the long scanning time enables the visualization of slow transversal blood flow in the deeper retinal capillaries, however on the other hand causes a fast saturation of the signal for increased blood flow^60^. This may be the reason why the signal in the deeper plexus vanishes earlier than in the superficial plexus as the slow blood flow cannot be visualized anymore in the latter case. A slight increase in the retinal flux with elevated IOP may be due a vasodilation effect of the capillaries as part of the autoregulatory process, which would also increase the number of flow-pixels in the angiogram. This trend is in agreement with the work of Wang *et al*. where a faint, although not statistically significant, increase of vessel diameter was observed in rats and monkeys with decreasing OPP^53^. One should mention that with increased IOP, the pulsatile motion increased and thus probably caused additional decorrelation artefacts^61^. A significant perfusion drop was observed for the retinal plexuses at 85 mmHg and 95 mmHg. In this high IOP regime, the IOP exceeds the diastolic blood pressure and blood flow stops, see Fig. 7(c). Although the blood pressure was not measured in this experiment, the pressure range is in good agreement with the physiological parameters found in literature for anesthetized rats^57^. In contrast to the retinal plexuses, the choroid showed rather continuous decrease in blood flux. This is likely to be related to the fact that at baseline the vessel density in the choroidal plexus is higher at baseline than in the retinal plexuses and thus small variations in the vessel area are less pronounced. Another reason could be that the autoregulative process is weaker in the choroid compared to the retina. Furthermore, some remaining blood vessels were still present in the choroid when retinal perfusion already had collapsed. Choroidal vessels are actually not expected to be perfused anymore at this high IOP level as the IOP exceeds the systolic blood pressure. However, due to heart beat activity some residual movement of the blood is still present which causes decorrelation of the OCT signal.

This IOP study has proven that physiological changes can be assessed by determining the fundus pulsatility in the rat eye using the proposed approach based on optical coherence based elastography providing high spatial resolution. In order to increase the accuracy of the proposed method, the experimental setting may be improved in future studies. For example, Moult *et al*. have shown that different anaesthesia protocols affect the stability of physiological parameters over time, e.g. the heart rate or the blood pressure, differently^57^. Thus, adapting the anaesthesia protocol and a more comprehensive evaluation of the midazolam/medetomidine/fentanyl anaesthesia protocol should be subject of future investigations. Furthermore, the cannulation of the eye results in some restrictions while handling the rats and a movement of the rat should be avoided. Thus, for example the contralateral eye, which may otherwise serve as a reference, could not be investigated^62^. The use of a vascular loop may be considered in the design of future studies using pressure elevation, as such a setup would also allow a precise measurement of the IOP at the eye using a pneumatonometer^39, 63^.

Previously used methods to determine ocular fundus pulsations such as laser interferometry or pneumotonometry lack the possiblity to map ocular fundus pulsation with high spatial resolution in a large FoV. Recent advances in OCT technology enabled the design of phase stable systems to detect fundus pulsation changes with high accuracy and simultaneously resolving surrounding ocular structures with high spatial resolution^21, 64^. The time-lapse between repeated B-scans enables axial displacement measurements in the nanometer range. Systems designated to detect ocular fundus pulsations focus on the detection of two or more ocular surfaces to eliminate bulk motion and hence enable the measurement of subtle distance changes following the principles used in laser interferometry to assess ocular distances. Singh *et al*. proposed an OCT system to detect the fundus pulsation amplitude between the cornea and the RPE in the retina^64^. Pulse induced changes were detected by Li *et al*. in the anterior segment of murine eyes between the cornea and the anterior lens surface^20^. Other approaches detected subtle deformations solely in the posterior eye without the need of a reference surface^19, 21^. For example, An *et al*. measured phase changes related to ONH pulsatilty by acquiring B-scans with a relatively large FoV of approximately 3 mm covering the ONH area as well as the peripapillary area serving as a reference to estimate the bulk motion^21^. O’Hara *et al*. investigated pulsatile deformations in the ONH and were able to detect the relative displacement and flexing of the lamina cribrosa^19^. In this work, we demonstrated measurements of pulse-induced deformations within the posterior albino rat eye between the retina and the chorioscleral complex, hereby using the signal in the retina as a reference to remove bulk motion. The lack of pigmentation in these animals enables deep penetration into the posterior eye. The increased heart rate in small animals such as mice and rats permits the inclusion of approximately 60–80 heart cycles in a 15 s acquisition period. Thus, we assured the sampling of sufficient heart cycles in neighbouring fundus regions while scanning around the ONH in two directions. Furthermore, we assumed that neighbouring regions in the fundus experience similar pulse-induced deformations. It should be mentioned that these assumptions may lead to an slight local under- or over-estimation of the actual deformation (averaging effect) in comparison to solely B-scan based methods.

Different intraocular absolute displacements and axial velocities have been described in the literature. Methods based on low-coherence tissue interferometry are typically reporting displacements between the cornea and the fundus in the range of 2 to 6 *μm* depending on the location^27, 29^. For example, Dragostinoff *et al*. report an average displacement of 1.7 *μm* and 2.0 *μm* at eccentricities of 0° and 7° relative to the optical axis, respectively^27^. These values were also confirmed in OCT measurements between the cornea and the retina by Singh *et al*.^28^. Deformations within the retina were described by O’Hara *et al*. where the flexing of the lamina cribrosa was measured to be in the range of 1 to 2 *μm* ^19^. An el al. reported axial velocities of the ONH in the range of −30 *μm*/*s* to 30 *μm*/*s* corresponding to an absolute displacement of approximately 3 *μm* ^21^. Within the anterior chamber of the mouse eye, Li *et al*. described displacements in the range of 180 nm^20^. In this work, absolute displacements in the range of 150 nm at physiological IOP of 15 mmHg to 300 nm at 65 mmHg were measured in the proximity of the ONH, see Fig. 3.

*In-vivo* measurements of ocular rigidity with OCT based elastography may have a high impact on the study of biomechanical properties in the multi-factorial pathophysiology of glaucoma. Hence, a translation of the proposed concept to the human eye may be exciting although some challenges have to be overcome. First of all, the relatively slow heart rate of approximately 1 Hz in humans requires rather long scanning times to cover at least a few cardiac cycles. Long scanning times are acceptable in animal experiments where animals are anaesthetized and fixed in a rodent stage but difficult to realize in patients, which may additionally have fixation disabilities. Hence, scanning times of 5–7 seconds may be the upper limit for patient measurements. Systems with an incorporated eye tracker, as used in commercial OCTA systems, may be implemented to target the problem of eye motion^65^. Furthermore, to ensure sufficient phase correlation and stability between successive frames, high imaging speeds are favorable in order to enable the calculation of subtle tissue deformations. Parallel methods such as full-field OCT may be beneficial and have already proven to be able to detect pulsatility and deformations induced by retinal blood vessel expansion with high temporal resolution^66^. Furthermore, the pigmentation in the RPE limits light penetration, especially in the most common commercial system wavelength range of 840 nm. However, swept source OCT operating at 1060 nm provides deeper tissue penetration and increased imaging speed and may therefore be a promising technology for the detection of tissue deformations and blood flow below the RPE. These latest developments in laser technologies may also be promising in detecting the choroidal blood flow which can probably reveal the key-factors in the multi-factoral pathogenesis of glaucoma using a multi-functional OCT approach^67–69^.

As limitations of the conducted experiment we want to mention that the number of rats investigated in this preliminary study was rather low and that the short-term increase of IOP limits the comparison of the measured parameters to studies of patients undergoing continuous impairment. Hence, to study the long-term impact of IOP on the physiology of the eye, a longitudinal investigation of the effect of elevated IOP in the posterior rat eye evaluated by multi-functional OCT should reveal more profound insights. Longitudinal changes of biomechanical properties could then be related to data of glaucomatous patients^29^. The proposed concept and the experimental evaluation revealed a substantial change in the fundus pulsatility as a function of IOP. The multi-functional concept of mapping OCTA data to ocular pulsatility simultaneously with high spatial resolution proved to be an exciting tool for studying various ocular parameters. The quantitative assessment of these parameters and experimental evaluations is promising in the study of multi-factorial pathophysiological processes of various diseases such as glaucoma.

## Electronic supplementary material

Time gated ocular fundus pulsations in response to elevated IOP.

Wide-field ocular fundus pulsations and OCTA.
